# Pathogenesis and potential relative risk factors of diabetic neuropathic osteoarthropathy

**DOI:** 10.1186/s13018-017-0634-8

**Published:** 2017-10-02

**Authors:** Hong-Mou Zhao, Jia-Yu Diao, Xiao-Jun Liang, Feng Zhang, Ding-Jun Hao

**Affiliations:** 1 Foot and Ankle Surgery Department, Honghui Hospital of Xi’an Jiaotong University College of Medicine, No. 76 Nanguo Road, Xi’an, 710054 People’s Republic of China; 2 Cardiovascular Medicine Department, The Second Affiliated Hospital of Xi’an Jiaotong University College of Medicine, No. 157 West Fifth Road, Xi’an, 710004 People’s Republic of China; 3 School of Public Health, Health Science Center Xi’an Jiaotong University, No. 76 Yan Ta West Road, Xi’an, 710061 People’s Republic of China; 4 Spine Surgery Department, Honghui Hospital of Xi’an Jiaotong University College of Medicine, No. 76 Nanguo Road, Xi’an, 710054 People’s Republic of China

**Keywords:** Charcot foot, Diabetic neuropathic osteoarthropathy, Pathogenesis, Risk factor, Receptor activator of nuclear factor κβ ligand (RANKL)

## Abstract

Diabetic neuropathic osteoarthropathy (DNOAP) is an uncommon, but with considerable morbidity and mortality rates, complication of diabetes. The real pathogenesis is still unclear. The two popular theories are the neuro-vascular theory and neuro-traumatic theory. Most theories and pathways focused on the uncontrolled inflammations that resulted in the final common pathway, receptor activator of nuclear factor κβ ligand (RANKL)/osteoprotegerin (OPG) axis, for the decreased bone density in DNOAP with an osteoclast and osteoblast imbalance. However, the RANKL/OPG pathway does not explain all the changes, other pathways and factors also play roles. A lot of DNOAP potential relative risk factors were evaluated and reported in the literature, including age, gender, weight, duration and type of diabetes, bone mineral density, peripheral neuropathy and arterial disease, trauma history, and some others. However, most of them are still in debates. Future studies focus on the pathogenesis of DNOAP are still needed, especially for the genetic factors. And, the relationship between DNOAP and those potential relative risk factors are still need to further clarify.

## Background

Musgrave firstly reported neuropathic joint changes as a complication of venereal disease in 1703 [1]. And neuropathic arthropathy is named after the French neurologist Jean-Martin Charcot (1825 to 1893), who firstly described the condition in 1868 as a complication of patients with tabes dorsalis [2]. However, the neuropathic inflammatory osteoarthropathy associated with the foot was firstly reported in 1881 by an English surgeon, Herbert William Page [3]. In 1936, Jordan was the first to describe Charcot in diabetes [4]. In more recent times, diabetes has become the most common etiology for the development of neuropathic arthropathy. Hence, the term diabetic neuropathic osteoarthropathy (DNOAP) has been used to describe such changes in the feet and ankles of patients with diabetes.

**Fig. 1:**
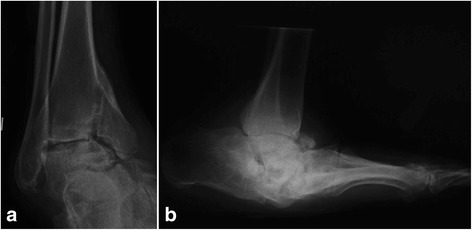
The radiographic characters of DNOAP. The fracture type (**a**) and the dislocation type (**b**)

DNOAP is a devastating complication for diabetes, culminating in bone destruction and involving joints and articular cartilage with high inflammatory environment and potentially leading to osteolysis and low bone mineral density, dislocations, fractures, and deformities (Fig. 1). The prevalence of DNOAP was reported from 0.08 to 13.0% in all patients with diabetes mellitus [5, 6], and the prevalence rise up to 29.0% in high-risk patients [7]. It was well established that DNOAP and relative complications severely reduces the overall quality of life and dramatically increases the morbidity and mortality of patients [8].

The etiology of DNOAP is supposed to be multifactorial. Two main theories concerning the origin of the condition are neuro-vascular theory and neuro-traumatic theory. However, classical etiological theories assessed the pivotal role of diabetic peripheral neuropathy (DPN), but clinical differences between DNOAP and DPN suggest that DPN is necessary but insufficient in explaining the pathogenesis of DNOAP [9]. Till now, the exact nature of DNOAP remains unknown. Many studies focused on the pathogenesis of DNOAP were published in recent two decades, and a lot of pathways were reported correlated with the development of DNOAP. A better understanding of the interplay between these complex pathways and common genetic polymorphisms among those affected by DNOAP is required to fully understand its pathogenesis [10–12]. A lot of DNOAP potential relative risk factors were reported in the literature; however, most of them are still in debates. In the current study, we give comprehensive review of the current updates of pathogenesis and those potential relative risk factors of DNOAP.

## Pathogenesis

### Neuro-vascular theories

The neuro-vascular theory, first described by Charcot, predicates a state of hyperemia generated from overactive vaso-autonomic neuropathy [13]. The increased blood flow increases venous pressure and enhances fluid filtration through capillary leakage [8]. This in turn leads to increased compartmental pressure and deep tissue ischemia. The increased pressure and ischemia compromises tendons and ligaments in the foot and ankle leading to joint instability [14]. Additionally, the increased blood flow may directly cause increased bone resorption by increasing the delivery of osteoclasts and monocytes resulting in greater osteoclastic activity in this area [15]. This is consistent with the finding that patients with a Charcot foot show increased blood flow to the area whereas patients with peripheral arterial disease and diabetes are relatively protected from developing the arthropathy [16, 17].

### Neuro-traumatic theory

Volkmann and Virchow proposed a neuro-traumatic theory for the pathogenesis of neuro-arthropathy [18]. This theory hypothesizes trauma (acute, subacute, or repetitive) as the causative factor in the setting of absent protective sensation. The bone and soft tissues respond with an acute-phase release of pro-inflammatory cytokines, tumor necrosis factor-α (TNF-α), interleukin-1β (IL-1β), and IL-6 [19]. And the cytokines upregulate the receptor activator of nuclear factor κβ ligand (RANKL) system. Study reported that after minor joint trauma, the progress of DNOAP might develop rapidly in weeks [20]. However, the neuropathic theory does not explain all the changes as only a small proportion of patients with neuropathy develop a Charcot foot [2, 18].

There is evidence that the nervous system and neuropeptides effect bone metabolism [21]. DNOAP showed higher degree of sympathetic and parasympathetic dysfunction than diabetic patients and normal control [22]. Sensory fibers contribute to the maintenance of trabecular bone integrity through mechanisms mediated by calcitonin gene-related peptide (CGRP) and/or substance P; in addition, ablation of CGRP results in osteopenia due to reduced osteoblastic bone formation [23, 24].

### Pro-inflammatory state

The pro-inflammatory state in diabetes is attributed to elevated concentrations of pro-inflammatory cytokines which are derived from increased protein kinase C (PKC) activity and advanced glycation end products (AGE)/receptor of AGE (RAGE) interactions accompanied by suppressed phosphatidylinositol 3 (PI3) kinase activity [25]. Also, repetitive or unrecognized trauma can trigger a cascade of inflammatory events. Munson et al. [26] concluded that identified novel associations with DNOAP in the context of pathogenesis models that include neurotrophic, neurovascular, and microtraumatic factors mediated through inflammatory cytokines.

The main pro-inflammatory cytokines, TNF-α, IL-1β, and IL-6, play a pivotal role in the inception of DNOAP via amplified RANKL expression and cause localized osteolysis that jeopardizes bone integrity making the high-risk diabetic foot vulnerable to ulceration, deformity, and fractures [27]. Meanwhile, there is a decrease in anti-inflammatory cytokines, IL-4 and IL-10, and osteoprotegerin (OPG) [19, 27–29]. Mabilleau et al. [19] found that the percentage of cluster of differentiation (CD) nomenclature 14 positive cells in acute DNOAP was significantly increased compared with diabetic and healthy controls, with a strong positive correlation to the TNF-α level.

### Oxidative stress

In DNOAP, an essential pathogenic pathway is considered to be a local dysregulation of immunoinflammatory processes, in which a principal role is played by oxidative stress and the formation of reactive oxidant species (ROS). Oxidative stress is associated with sequential oxidative reactions, which generate AGEs that may cause oxidative post-translational modification (oxPTM) of tissue’s proteins [30]. Rizzo et al. [9] detected the presence of autoantibodies against oxPTM collagen, particularly type 2 collagen in participants with DNOAP, suggesting the possible involvement of autoimmunity. Comparing the diabetic patients with or without DNOAP and the healthy controls showed a different expression of the serum levels of antioxidants in all three groups, with the lowest expression in the DNOAP group [2].

### RANKL/OPG pathway

The most accepted pathway is the RANKL/OPG axis. RANKL is a member of the TNF superfamily. OPG is the competitive protein of RANKL and antagonizes the pathway. The system is a key mediator of bone metabolism, and it has been used to evaluate osteoclastogenesis and osteolytic processes in a number of disease states such as rheumatoid arthritis, osteoarthritis, bone tumors, prosthetic failure, and periodontal disease [31]. Sinacore et al. [32] reported that the local and systemic inflammation persists 1 year after DNOAP with an accompanying pedal osteolysis. The RANKL/OPG pathway seems to be central to the development of DNOAP with prolonged inflammation, and osteolysis causes microfractures and local bone destruction [2].

The process of bone resorption and formation is controlled by the level of RANKL and OPG, and many factors may contribute to this pathway. Hyperglycemia may increase the level of AGEs, ROS, and oxidized lipids, which may all enhance the expression of RANKL in diabetes [22, 33, 34]. The pro-inflammatory state increases cytokines’ expression, TNF-α, IL-1β, and IL-6, which may also enhance the expression of RANKL [19, 28, 29, 35]. Diabetic neuropathy is associated with exhaustion of CGRP stores from C and Aδ nerve fibers. The deficiency of CGRP may further accelerate the underlying osteopenia due to unrestricted RANKL activity [36]. RANKL has been shown to mediate osteolysis in DNOAP by stimulating osteoclastic differentiation of monocytes/macrophages and trigger the synthesis of the nuclear transcription factor, nuclear factor-κβ (NF-κβ), to stimulate the maturation of osteoclasts from osteoclast precursor cells [28, 37]. A repetitive trauma with the loss of pain sensation results in continual production of pro-inflammatory cytokines, RANKL, NF-κβ, and osteoclasts, which in turn leads to continuing local osteolysis [33].

Under inflammatory conditions, both B and T cells can be considerable sources of RANKL and may contribute to pathological bone resorption [38, 39]. However, in acute DNOAP, the local inflammatory response related to increased pro-inflammatory cytokine secretion is not associated with a systemic inflammatory syndrome [40]. Bergamini et al. [41] reported that the RANKL expression was significantly lower in the peripheral blood mononuclear cells of acute DNOAP patients than that in normal and diabetic controls and the RANKL expression increased at the time of healing compared with the values of acute phase. Their data suggested the existence of compensatory immunoregulatory mechanisms to potentially limit bone resorption during acute DNOAP.

### AGE/RAGE pathway

Hyperglycemia generates AGEs which occur from non-enzymatic reactions between glucose and glycating compounds with proteins. The formation of AGEs elevated during the earliest detectable phase of DNOAP [34], and it is an important biochemical abnormality that accompanies diabetes and inflammation in general [22]. AGEs promote irreversible posttranslational modification of both intracellular and extracellular proteins causing these proteins to lose their functionality and become defective [25]. The RAGE was reported being displayed more than seven times lower in DNOAP patients than in control subjects and more than three times lower than in diabetic patients [22]. Binding of AGE to RAGE expressed over macrophages accelerates ROS production which activates NF-κB. Increased NF-κβ activity induces pathologic gene expression leading to generation of IL-1, TNF-α, transforming growth factor-β (TGF-β), and macrophage colony-stimulating factor. An increase in AGE-modified collagen affects osteoblastic cell differentiation and function in vitro and may play a role in the pathogenesis of osteopenia present in patients whose diabetes is poorly controlled [42]. AGEs also stimulate apoptosis of human mesenchymal stem cells and osteoblast apoptosis through a NF-κβ independent mechanism that further limits bone formation.

### PI3 kinase pathway

PI3 kinase normally impedes inflammation through enhanced nitric oxide (NO) production and restrained expression of adhesion molecules. Several studies suggest that NO has a reciprocal effect on the modulation of bone metabolism [24]. NO was reported being able to induce apoptosis of pre-osteoclasts and decrease the resorptive action of the mature osteoclasts in animal studies [43]. PI3 kinase defense resulted in the decrease of NO production and is compromised in diabetes, enabling progression of endothelial dysfunction [25].

### PKC pathway

PKC, a pro-inflammatory pathway, occurs from accumulation of diacylglycerol, a glucose intermediate derived from hyperglycemia. The pathologic sequelae that follow PKC activation include decreased NO bioavailability through suppression of endothelial NO synthase (eNOS), amplified NF-κβ expression, increased endothelin-1, enhanced plasminogen activator inhibitor-1 activity, and ROS production [24, 44].

### Wnt/β-catenin pathway

A recent study found that the levels of sclerostin, dickkopf-1 (Dkk-1), and Wnt ligand-1 (Wnt-1) were significantly lower in acute DNOAP patients than in diabetic and healthy controls and concluded that the Wnt/β-catenin pathway, an important bone anabolic pathway [45], has a role in bone remodeling and bone repair activity in DNOAP patients [46]. Earlier study showed that inhibition of Dkk-1 and sclerostin improved healing of fractured bone [47]. However, other study further evaluated the role of Wnt/β-catenin pathway in DNOAP.

### Role of FLS

Fibroblast-like synoviocytes (FLS) line on the intimal lining layer of the synovium. When activated by inflammatory cytokines, FLS secrete prodigious amounts of matrix metalloproteinases (MMPs) responsible for degrading articular cartilage [48]. Molligan et al. [49] reported that FLS may play an important role in the joint destruction in DNOAP. The nerve fibers in DNOAP synovium were significantly less than in normal control. The expression cadherin-11, as well as ADAMTS-4 (a disintegrin and metalloproteinase with thrombospondin motifs 4), ADAMTS-5, and IL-6, were increased in DNOAP patients, and the RANKL was upregulated [49]. However, the pathogenesis study focused on the joint destruction is still very limited.

### Osteoprotegerin gene polymorphism

Recently, an association between two OPG polymorphisms (1181G>C and 245T>G) and DNOAP was suggested [50] and was the first report on a possible contribution of OPG gene polymorphism to this diabetic complication. Korzon-Burakowska et al. [11] reported the significant differences between DNOAP patients and normal control for 1181G>C and 950T>C polymorphisms and between DNOAP patients and neuropathy patients for 1217C>T and 245T>G polymorphisms. And they suggested that genetic factors, particularly OPG gene polymorphisms, may play a role in the development of DNOAP [11]. These findings open new perspectives for improved understanding and further research in the field of pathophysiology, but confirmation in other populations is still awaited.

## Relative risk factors

### Age, gender, weight

Age is an associated risk factor with DNOAP, but in debates. Petrova et al. [51] reported the Charcot neuropathy typically presents in patients with diabetes during the fifth or sixth decade of life. Other studies report the most patients with DNOAP in their sixth and seventh decades [2, 52]. Fauzi et al. [16] and Nobrega et al. [17] reported that age below 60 and 55 years were significant risk factors for DNOAP respectively.

Most studies showed the ratio of DNOAP in men and women was approximately the same and no definite sex predilection [5]. However, Nehring et al. [53] reported that male gender was more likely to suffer from DNOAP.

Whether weight is associated with increased risk of DNOAP was in debate. Some studies reported positive correlations [53–55]. However, no association was found in other studies between DNOAP and a body mass index more than 25 kg/m^2^ [7, 16]. Stuck et al. [54] concluded that weight loss could significantly reduce the risk of DNOAP, but might be most effective for patients with peripheral neuropathy.

### Duration and type of diabetes

Most studies confirmed that the diabetes duration was a risk factor to DNOAP occurrence [51, 55, 56]. However, there are type differences in the demographic features of patients with type 1 and type 2 diabetes developing the DNOAP [51]. Patients with type 1 had a longer duration of diabetes than those with type 2, but developed Charcot at an earlier age. Petrova et al. [51] included 83 patients, 44 with type 1 diabetes presented DNOAP mostly in their fifth decade (40–49 years), while 41 type 2 patients in their sixth decade (50–59 years); however, the type 1 patients developed DNOAP with a significantly longer duration than those with type 2 diabetes (24 ± 8.4 vs. 13 ± 8.1 years). Pakarinen et al. [55] reported 36 patients, and the average duration of type 1 was 28 years and that of type 2 was 14 years.

### Bone mineral density

The relationship between bone mineral density (BMD) and DNOAP is unclear. It is unknown whether regional osteopenia is a result of the inflammatory process that accompanies the bone injury or is a risk factor for developing neuropathic joint disorders. Jones et al. [57] reported primary resorption of bone without subluxation, dislocation, and/or fracture in the active Charcot process. Nonetheless, osteopenia, reduced bone stiffness, and decreased BMD have been shown radiographically in acute and chronic DNOAP patients [22, 32, 51, 58, 59]. Sinacore et al. [60] reported that inflammation in DNOAP may contribute to or exacerbate a rapid loss of BMD.

The relation between BMD loss and immobilization or off-loading is in debate. Some studies reported the BMD may further loss during casting and no weight bearing [58]. However, Pakarinen et al. [61] reported that immobilization and off-loading does not lead to marked disuse osteoporosis in patients with acute DNOAP after 6 months of treatment. BMD of DNOAP is also different according to the pattern of initial destruction. Herbst et al. [59] divided 61 Charcot feet or ankles into three subtypes, fracture pattern, dislocation pattern, and combined fracture-dislocation pattern, and found that the fracture pattern was associated with a peripheral deficiency of BMD, while the dislocation pattern was not.

### Peripheral neuropathy and arterial disease

Peripheral neuropathy is associated with all disorders that produce neuroarthropathy. Just as mentioned above, severe peripheral neuropathy typically creates a loss in protective sensation, progressing the development of DNOAP. As reported by Fabrin et al. [62], 100% of included DNOAP patients had peripheral neuropathy as determined by clinical exam and biothesiometer.

Peripheral arterial disease seems have no relationship with DNOAP. Compared with patients with diabetic foot ulceration, the patients with DNOAP were less likely to have peripheral arterial disease, ischemia, or the need for revascularization [63]. Nobrega et al. [17] also reported that peripheral arterial disease was a protective factor to DNOAP.

### Trauma history

Diabetic neuropathy and decreased BMD make the insensate foot vulnerable to trauma. The initial bone injury is usually subtle and unrecognized by the neuropathic patient. Repetitive or unrecognized trauma, including foot surgery history, can trigger a cascade of inflammatory events and may lead to irreversible destruction of bone microarchitecture and ligament instability [64]. In an audit on acute DNOAP in UK and Ireland [65], 36% of patients reported some trauma and 12% reported foot surgery during the preceding 6 months. A pooled result showed that the preceding trauma may be recalled in as many as half of all cases of acute DNOAP (25–50%) [56]. And in incidences where no trauma is recalled, repetitive microtrauma on an insensate foot may be a contributing factor [7, 55].

### Other potential relative risk factors

An increased rate of DNOAP has been noted in simultaneous pancreas-kidney transplant (SPKT) patients. Matricali et al. [66] reports 12% of 66 patients developed neuropathic joints post-transplant, and four of them presented within 1 year following transplantation. Garcia Barrado et al. [67] evaluated 100 SPKT patients with type 1 diabetes and without previous Charcot arthropathy; 9% developed DNOAP after transplantation, and four presented within 1 year. And the patients with DNOAP after SPKT have a significantly higher mortality rate (56 vs. 18%) and kidney graft failure rate (44 vs. 13%) [67].

Other potential relative risk factors reported in the literature including increased levels of HbA1c in serum, treatment with insulin/insulin and oral hypoglycemic agents, retinopathy, prolonged walking, living alone, and low literate education [16, 17, 22, 54, 66, 68].

As we all know, not all of the diabetic patients develop DNOAP. However, the patients combined with one or more potential risk factors may increase the probability of having DNOAP. Some factors are controllable and treatable, such as low BMD, high body mass index, and trauma; doctors should tell the diabetic patients to improve the body conditions and to take care of bony or soft tissue trauma in their daily life. But some factors are uncontrollable and untreatable, such as duration and type of diabetes, age, gender, trauma history, and SPKT. The doctors should follow these patients closely and give early diagnosis and necessary treatment, such as total contact cast to prevent the development of fracture or dislocation.

## Conclusions

**Fig. 2:**
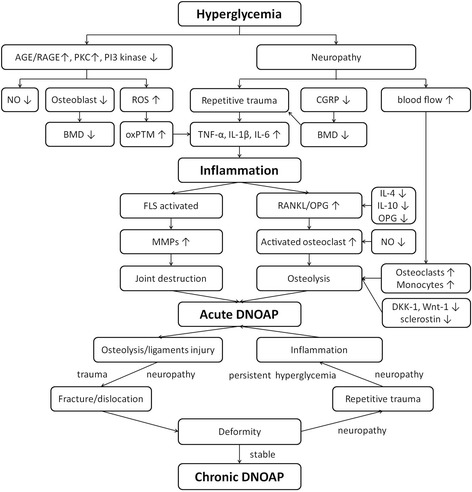
The pathogenesis of DNOAP. DNOAP diabetic neuropathic osteoarthropathy, AGE advanced glycation end products, RAGE receptor for advanced glycation end products, PKC protein kinase C, PI phosphatidylinositol, NO nitric oxide. ROS reactive oxidant species, CGRP calcitonin gene-related peptide, BMD bone mineral density, oxPTM oxidative post-translational modification, IL interleukin, TNF tumor necrosis factor, FLS fibroblast-like synoviocytes, RANKL receptor activator of nuclear factor κβ ligand, OPG osteoprotegerin, MMPs matrix metalloproteinase, Dkk-1 dickkopf-1, Wnt-1 Wnt ligand-1

In summary, DNOAP is an uncommon complication of diabetes but is potentially devastating in its consequences. Some theories and pathways were developed to explain the pathogenesis and mostly focused on the uncontrolled inflammations that result in the final common pathway for the decreased bone density in DNOAP with an osteoclast and osteoblast imbalance. And the inflammatory condition may be further enhanced by the neuropathy, peripheral arterial disease, painless ambulation, and unrecognized trauma (Fig. 2). Studies focused on the DNOAP joint and soft tissue destruction are very limited. The DNOAP potential relative risk factors may accelerate the progression of the pathological changes. However, the real pathogenesis is still unclear, and the most of relative risk factors are still in debate. Further studies focused on the pathogenesis and relative risk factors are still needed to clear the development of this disabling complication.

## Abbreviations

ADAMTS-4: A disintegrin and metalloproteinase with thrombospondin motifs
AGE: Advanced glycation end products
BMD: Bone mineral density
CD: Differentiation nomenclature
CGRP: Calcitonin gene-related peptide
Dkk-1: Dickkopf-1
DNOAP: Diabetic neuropathic osteoarthropathy
DPN: Diabetic peripheral neuropathy
eNOS: Endothelial NO synthase
FLS: Fibroblast-like synoviocytes
IL: Interleukin
MMPs: Matrix metalloproteinases
NF-κβ: Nuclear factor-κβ
NO: Nitric oxide
OPG: Osteoprotegerin
oxPTM: Oxidative post-translational modification
PI: Phosphatidylinositol
PKC: Protein kinase C
RAGE: Receptor for advanced glycation end products
RANKL: Receptor activator of nuclear factor κβ ligand
ROS: Reactive oxidant species
SPKT: Simultaneous pancreas-kidney transplant
TGF-β: Transforming growth factor-β
TNF: Tumor necrosis factor
Wnt-1: Wnt ligand-1
